# A Prospective Study of Frailty and 30-Day Functional Outcomes of Hospitalized Older Adults With Pneumonia

**DOI:** 10.1093/geroni/igaa057.442

**Published:** 2020-12-16

**Authors:** Chan Mi Park, Wonsock Kim, Jong Hun Kim, Dae Kim

**Affiliations:** 1 Korea University Anam Hospital, Seoul, Republic of Korea; 2 Hebrew SeniorLife, Harvard Medical School, Roslindale, Massachusetts, United States

## Abstract

Pneumonia is a major cause of morbidity and mortality in older adults. The role of frailty assessment in older adults with pneumonia is not well defined. A prospective cohort study was conducted to investigate the clinical and functional outcomes of pneumonia in older adults with different levels of frailty. Between November 2019 and March 2020, we enrolled 92 adults who were 65 years or older and hospitalized for pneumonia at an academic medical center in Seoul, Korea. A deficit-accumulation frailty index (FI) was calculated using 50 items from comprehensive geriatric assessment (range: 0-1; higher values indicate greater frailty). The ability to perform 21 daily activities and physical tasks prior to the illness and after 30 days was self-reported. Primary outcome was death or functional decline from prior level at 30 days. The study cohort had a mean age of 79.4 (standard deviation [SD], 7.11) years, 38 (41.3%) women, and 20 (21.7%) nursing home residents. The mean FI was 0.29 (SD, 0.19), with 27 (29.4%) robust (FI<0.15), 21 (22.8%) pre-frail (FI, 0.15-0.24), 19 (20.7%) mild-to-moderately frail (FI, 0.25-0.44), and 25 (27.2%) severely frail (FI≥0.45) categories. Among 71 patients without maximum disability at baseline, 8 (11.3%) died and 39 (54.9%) experienced functional decline. The 30-day risk of primary outcome for increasing frailty categories were 47.8%, 57.9%, 77.8%, and 81.8%, respectively (p-for-trend=0.022). Our results indicate that pneumonia is a major disabling illness in older adults with frailty. A proactive geriatric evaluation and multidisciplinary intervention are needed to improve functional recovery in these patients.

